# Effects of *Bifidobacterium breve* B-3 on Glycemic Control and Glucose-Regulatory Responses in db/db Mice

**DOI:** 10.4014/jmb.2605.05040

**Published:** 2026-08-18

**Authors:** Sungmin Cho, Yewon Jeong, Jae-Hoon Kim, Yuri Gwon, Hakdong Shin, Wonchul Lim, Tae-Gyu Lim

**Affiliations:** 1 Department of Food Science & Biotechnology, Sejong University, Seoul 05006, Republic of Korea; 2 R&D Division, Daehan Chemtech Co., Ltd., Gwacheon, Republic of Korea; 3 Department of Food Science & Biotechnology, and Carbohydrate Bioproduct Research Center, College of Life Science, Sejong University, Seoul 05006, Republic of Korea

**Keywords:** *Bifidobacterium breve* B-3, Probiotics, Glycemic control, GLP-1, Glucose metabolism, db/db mice

## Abstract

Type 2 diabetes mellitus is characterized by chronic hyperglycemia and impaired glucose homeostasis. This study evaluated the anti-diabetic effects of *Bifidobacterium breve* B-3 using *in vitro* models and db/db mice. *B. breve* B-3 inhibited α-amylase activity and enhanced insulin-stimulated glucose uptake in palmitic acid-induced insulin-resistant C2C12 myotubes. Metabolite profiling also showed distinct strain-associated metabolic characteristics of *B. breve* B-3. In db/db mice, oral administration of *B. breve* B-3 significantly reduced fasting blood glucose levels and improved oral glucose tolerance without affecting body weight. These effects were accompanied by changes in incretin-related endocrine responses, including increased serum GLP-1 levels and decreased DPP-4 activity. *B. breve* B-3 also increased serum insulin and C-peptide levels and partially preserved pancreatic insulin immunoreactivity. In addition, mild diabetes-associated intestinal morphological alterations and glucose-regulatory marker expression were partially normalized after administration. In skeletal muscle, *B. breve* B-3 was associated with increased expression of glucose uptake- and metabolic signaling-related markers, including AKT/GLUT4 and ADIPOR1/AMPK pathways. Overall, these findings suggest that *B. breve* B-3 improves glycemic control in db/db mice in association with changes in incretin-related endocrine responses and glucose-regulatory signaling markers.

## Introduction

Diabetes mellitus is a major global metabolic disorder characterized by chronic hyperglycemia resulting from impaired insulin secretion, insulin action, or both [1]. Type 2 diabetes mellitus (T2DM) accounts for the majority of cases and is strongly associated with obesity, chronic low-grade inflammation, and insulin resistance [2, 3]. Persistent hyperglycemia contributes to multi-organ complications, including hepatic, renal, muscular, and cardiovascular dysfunction [4]. Although various pharmacological therapies are available, long-term glycemic control remains suboptimal due to limited efficacy, adverse effects, and poor compliance [5, 6]. Accordingly, nutritional and microbiome-based strategies have emerged as complementary approaches for T2DM management [7].

The gut microbiota plays a critical role in metabolic homeostasis, and dysbiosis has been implicated in impaired gut barrier function, endotoxemia, systemic inflammation, and insulin resistance [8]. In contrast, modulation of gut microbiota through probiotics or prebiotics has been reported to improve glucose and lipid metabolism and to influence glucagon-like peptide-1 (GLP-1)-related endocrine responses through microbial metabolites, including short-chain fatty acids [9, 10]. These findings support the potential of probiotics as dietary interventions for metabolic disorders, including type 2 diabetes mellitus [11].

T2DM is characterized by dysregulation of multiple pathways governing glucose homeostasis [12]. Intestinal glucose absorption is primarily mediated by sodium-glucose cotransporter-1 (SGLT1) and glucose transporter-2 (GLUT2) and altered expression of these transporters has been associated with postprandial glucose dysregulation under diabetic conditions [13, 14]. Incretin signaling constitutes another central regulatory axis, in which GLP-1 enhances glucose-dependent insulin secretion, while dipeptidyl peptidase-4 (DPP-4) limits incretin activity through GLP-1 degradation [15]. In addition, short-chain fatty acid-responsive receptors, including GPR41 and GPR43, have been associated with metabolic and endocrine regulation [16, 17]. Beyond the intestine, impaired insulin signaling in skeletal muscle, characterized by attenuated AKT activation together with dysregulation of AMP-activated protein kinase (AMPK), contributes to reduced glucose uptake and systemic metabolic dysregulation in T2DM [18, 19]. As insulin resistance progresses, compensatory hyperinsulinemia and β-cell dysfunction contribute to persistent hyperglycemia and metabolic dysregulation in T2DM [20]. Collectively, disruption of these interconnected signaling pathways represents a central feature of T2DM pathophysiology [21].

Among beneficial gut microbes, *Bifidobacterium* species are well-established contributors to intestinal and metabolic health [22]. They regulate host metabolism through carbohydrate fermentation, suppression of pathogenic bacteria, and immune modulation [23]. Several *Bifidobacterium* strains have been reported to attenuate obesity and insulin resistance in both clinical and preclinical studies [24]. Notably, *Bifidobacterium breve* B-3 has demonstrated anti-obesity and metabolic benefits, including reductions in adiposity, attenuation of low-grade inflammation, and improvements in metabolic homeostasis [25]. However, mechanistic evidence linking *B. breve* B-3 to glucose-regulatory signaling pathways remains limited, as previous studies have primarily focused on phenotypic outcomes. Probiotic efficacy is generally strain- and disease-context dependent [26]. Therefore, *Lactobacillus plantarum* SJ was included as a single non-*Bifidobacterium* probiotic comparator rather than as a positive control for glycemic improvement. This comparator was used to contextualize whether the effects of *B. breve* B-3 reflected a general probiotic-associated response or a *Bifidobacterium*-associated functional profile under the present experimental conditions.

Therefore, the present study investigated the glycemic-regulatory effects of *B. breve* B-3 in db/db mice, a well-established model of type 2 diabetes [27, 28]. Glycemic parameters, including fasting blood glucose and oral glucose tolerance, were evaluated along with circulating insulin, GLP-1, and DPP-4 activity [29, 30]. In addition, intestinal expression of glucose transporters and short-chain fatty acid-responsive receptors was analyzed, together with insulin-related signaling in skeletal muscle to explore potential mechanisms associated with improved glycemic regulation [18, 31]. Overall, this study provides evidence that *B. breve* B-3 improves glycemic parameters and is associated with modulation of glucose-related signaling pathways in db/db mice, supporting its potential as a functional probiotic for metabolic health.

## Materials and Methods

### Reagents and Antibodies

3-(4,5-Dimethylthiazol-2-yl)-5-(3-carboxymethoxyphenyl)-2-(4-sulfophenyl)-2H-tetrazolium (MTS) (CellTiter 96^®^ AQueous Non-Radioactive Cell Proliferation Assay) solution was purchased from Promega (USA). Metformin (Met) was purchased from Sigma-Aldrich (USA). Monoclonal antibody against Vinculin was purchased from Santa Cruz Biotechnology (USA). Antibodies against AKT, phospho-AKT, AMPK, and phospho-AMPK were obtained from Cell Signaling Technology (USA), and GLUT4 antibody was purchased from Bioss Antibodies Inc. (USA).

### Preparation of *Bifidobacterium breve* B-3 and *Lactobacillus plantarum* SJ

Lyophilized *B. breve* B-3 and *L. plantarum* SJ powders were provided by Daehan Chemtech Co., Ltd., (Republic of Korea). *B. breve* B-3 and *L. plantarum* SJ were suspended in saline just before daily administration.

### Culture Conditions and Preparation of Cell-Free Supernatants

*B. breve* B-3 and *L. plantarum* SJ were cultured in MRS broth (55 g/L, Difco/BD) supplemented with 0.05% (w/v) L-cysteine. *L. plantarum* SJ was incubated at 37°C for 24 h under aerobic conditions, whereas *B. breve* B-3 was incubated at 37°C for 24 h under anaerobic conditions. After incubation, bacterial cultures were centrifuged at 4,000 × g for 10 min at 4°C to remove bacterial cells. The collected supernatants were filtered through a 0.22 μm membrane filter and stored at -80°C until further analysis.

### α-Amylase Inhibitory Activity Assay

α-Amylase inhibitory activity was evaluated using an Alpha-Amylase Inhibitor Screening Kit (Colorimetric) (Abcam, UK; ab283391) according to the manufacturer’s instructions. The cell-free culture supernatant (CFS) of *B. breve* B-3 was used as the test sample and was diluted to the indicated CFS levels using the assay buffer provided in the kit. After incubation with α-amylase and chromogenic substrate, absorbance was measured at 405 nm. Percent inhibition was calculated relative to enzyme control according to the manufacturer’s protocol.

### Cell Culture and Treatment

Murine skeletal muscle C2C12 myoblasts were cultured in Dulbecco’s modified Eagle’s medium (DMEM) supplemented with 10% fetal bovine serum and 1% penicillin–streptomycin at 37°C in a humidified atmosphere containing 5% CO_2_. For differentiation into myotubes, cells were grown to 80–90% confluence and subsequently maintained in DMEM containing 2% horse serum for 6 days, with media replaced every 2 days. To induce insulin resistance, differentiated myotubes were treated with 0.5 mM palmitic acid (PA) conjugated with 0.5% fatty acid–free bovine serum albumin (BSA) in serum-free DMEM for 24 h. PA was prepared as a 100 mM stock solution in ethanol and diluted into pre-warmed BSA-containing medium to achieve the desired concentration. The final ethanol concentration was adjusted to 0.5% (v/v), and vehicle control media contained an equivalent concentration of ethanol.

### Glucose Uptake Assay

Glucose uptake was evaluated in differentiated C2C12 myotubes using the fluorescent glucose analog 2-N-(7-nitrobenz-2-oxa-1,3-diazol-4-yl) amino-2-deoxyglucose (2-NBDG). C2C12 myoblasts were seeded in 96-well plates at 5.0 × 10^3^ cells/well and differentiated in DMEM containing 2% horse serum for 6 days. After serum starvation, myotubes were treated with 0.5 mM palmitic acid and lyophilized *B. breve* B-3 sample at 50–1600 μg/mL for 24 h. Cells were then stimulated with insulin (100 nM) for 30 min and incubated with 2-NBDG at 37°C for 30 min. After washing with phosphate-buffered saline, intracellular fluorescence was measured using a microplate reader at excitation/emission wavelengths of 485/535 nm. Relative glucose uptake activity was expressed as a percentage of the untreated control group.

### U-HPLC MS/MS Analysis for Metabolite Profiling

Metabolite profiling was performed using an ultra-high-performance liquid chromatography (U-HPLC) system (Vanquish^TM^ U-HPLC, Thermo Fisher Scientific, USA), coupled to a quadrupole mass spectrometer, (Thermo Scientific Orbitrap Exploris^TM^ 120 high-resolution/accurate mass spectrometer, equipped with a heated electrospray ionization (H-ESI) source), at the Biopolymer Research Center for Advanced Materials (BRCAM, Sejong University, Republic of Korea). PDA detection was performed at 210 nm in 3D scan mode. Cell-free culture supernatant samples were filtered through a 0.22 μm membrane filter prior to analysis. Briefly, 10 μL of each sample was injected into a C18 column (Waters, ACQUITY UPLC BEH C18, 2.1 × 100 mm, 1.7 μm, Waters Corp., USA), with the column maintained at 45°C throughout the acquisition period. The mobile phase was eluted by a gradient of water (A) and acetonitrile (B), containing 0.1% acetic acid, with a gradient dilution profile of 99% A (0–2 min), 99–5% A (2–20 min), 5% A (22–25 min), 5–99% A (25–27 min), and 99% A (27–30 min), at a flow rate of 0.25 mL/min. The mass spectrum conditions included a heated capillary of 325°C, a vaporizer temperature of 350°C, a spray voltage of 3.5 kV in positive mode, and 2.5 kV in negative mode; the sheath gas, aux gas, and sweep gas were set at 50, 25, and 1 psi, respectively. The HCD collision energy was set at 15%, 30%, and 60%, and the scan range was set to 100–1500 m/z in full scan mode, with a resolution of 120,000 for MS1 and 15,000 for MS2. The raw MS data were processed using Xcalibur version 4.6 and Compound Discoverer 3.3 (Thermo Fisher Scientific). Compounds were identified by matching m/z values and MS/MS fragments against the mzCloud and Chemspider databases.

### Metabolic Pathway and Interaction Network Analysis

Untargeted metabolomic data were subjected to pathway enrichment analysis using MetaboAnalyst (version 6.0) [32]. Differential metabolites between *B. breve* B-3 and *L. plantarum* SJ were selected based on fold change, and pathway analysis was performed using the Kyoto Encyclopedia of Genes and Genomes (KEGG) database. Pathway analysis was performed using topology-based enrichment analysis and over-representation analysis. To further explore the potential interaction context of strain-enriched metabolites, exploratory interaction networks were generated using the STITCH database (version 5.0) [33]. Interaction networks were generated based on known and predicted associations derived from curated databases, experimental data, and text mining sources. The organism setting was restricted to Homo sapiens to explore potential associations of strain-enriched metabolites with human metabolic pathways.

### Animal Experiments

All animal experiments were approved by the Animal Experimentation Committee of Sejong University (Approval No. SJ-20250415-03). Five-week-old male db/db and heterozygous db/+ mice (BKS.Cg-Dock7m +/+ Leprdb/db/J and BKS.Cg-Dock7m +/+ Leprdb/+ /J; Jackson Laboratory nomenclature) were acclimated for one week prior to experimentation (Orient Bio Inc., Seongnam, Republic of Korea). Mice were maintained under controlled environmental conditions (22 ± 2°C, 50 ± 10% humidity, 12 h light/dark cycle). After acclimation, mice were randomly divided into six groups (n = 10 per group): non-diabetic control (db/+, NC), diabetic control (db/db), *B. breve* B-3 low-dose (B-3 L), *B. breve* B-3 high-dose (B-3 H), *L. plantarum* SJ (Lacto), and metformin (Met). B-3 L was orally administered as a suspension containing 1 × 10^8^ colony-forming units (CFU)/mL in a volume of 200 μL, while B-3 H and *L. plantarum* SJ were administered at 1 × 10^9^ CFU/mL in the same volume. Metformin was administered at 250 mg/kg body weight. All samples were freshly prepared in 0.9% NaCl solution and administered orally once daily, five times per week for ten weeks. Body weight, food intake, and water consumption were recorded weekly, while fasting blood glucose levels were measured weekly.

### Fasting Blood Glucose (FBG) and Oral Glucose Tolerance test (OGTT)

For FBG measurement, mice were fasted for 8 h (23:00–07:00) once weekly for 10 weeks. The tail vein was disinfected with an alcohol swab, and a small incision was made at the tip. The first drop of blood was discarded, and the second drop was used for glucose measurement using a glucometer (Contour, Switzerland). After 10 weeks of intervention, all mice were fasted for 8 h, and an OGTT was performed by orally administering glucose (2 g/kg body weight, 20% w/v solution). Tail blood samples were collected at 0, 15, 30, 60, 90, and 120 min after glucose administration, and blood glucose levels were determined using the same glucometer. The area under the glucose curve (AUC) for blood glucose levels from 0 to 120 min was calculated to evaluate glucose tolerance.

### Serum Analysis

Following an overnight fasting period (approximately 12 h), mice were euthanized, and blood samples were collected at the time of sacrifice. Blood samples were transferred into serum separation tubes (SST; BD Vacutainer, Becton Dickinson, USA) and allowed to stand at room temperature for 30 min to facilitate clot formation. The coagulated samples were then centrifuged at 3,000 × g for 15 min, and serum was collected for subsequent analyses. Additional serum biochemical analyses, including alanine aminotransferase (ALT), aspartate aminotransferase (AST), and alkaline phosphatase (ALP), were conducted at Dooyeol Biotech (Seoul, Korea) using a BC-5000 Vet Auto Hematology Analyzer (Mindray, China). HOMA-IR was calculated using the following formula: fasting glucose (mg/dL) × fasting insulin (ng/mL) × 29 / 405, and was used as an indirect index of fasting glucose–insulin homeostasis.

### Quantification of Serum GLP-1, C-Peptide, Insulin, and Adiponectin

Serum concentrations of total GLP-1, C-peptide, insulin, and adiponectin were quantified using commercial ELISA kits according to the manufacturers’ instructions. Specifically, the following kits were used: Multi Species GLP-1 ELISA Kit (total; Millipore, USA), Mouse C-Peptide ELISA Kit (Crystal Chem, USA), Ultra-Sensitive Mouse Insulin ELISA Kit (Crystal Chem), and Mouse Adiponectin ELISA Kit (Crystal Chem). All measurements were performed in duplicate using serum samples obtained from each mouse, and absorbance was measured at 450 nm using a Cytation 1 microplate reader (BioTek, USA) at the Biopolymer Research Center for Advanced Materials (BRCAM). Concentrations were calculated from standard curves generated for each assay.

### Determination of DPP-4 Activity in Serum

Serum DPP-4 activity was determined using a fluorometric assay kit (Abcam, UK) following the manufacturer’s protocol. Briefly, serum samples were incubated with a specific DPP4 substrate, and enzymatic activity was quantified by measuring fluorescence intensity (Ex/Em = 360/460 nm) using a Cytation 1 microplate reader (BioTek, USA) at the Biopolymer Research Center for Advanced Materials (BRCAM).

### Quantitative Real-Time PCR

Total RNA was isolated from small intestine tissue using TRIzol reagent (Thermo Fisher Scientific). Complementary DNA (cDNA) was synthesized using the cDNA Synthesis Platinum Master Mix (GenDEPOT, USA). Quantitative real-time PCR (qRT-PCR) was performed using a SYBR Green-based detection system to evaluate gene expression levels. The primer sequences used for qRT-PCR are listed in Table 1. Relative gene expression levels were normalized to *β-actin* and calculated using the 2^-ΔΔCt^ method.

### Histological Analysis

Small intestine and pancreas tissues were harvested from mice at the end of the experiment and fixed in 4% formaldehyde. The fixed tissues were embedded in paraffin, sectioned at a thickness of 5 μm, and mounted onto glass slides. For histological evaluation of intestinal morphology, paraffin-embedded small intestine sections were stained with hematoxylin and eosin (H&E) according to standard procedures. General villus morphology and histological features were comparatively evaluated among experimental groups. For immunohistochemical analysis, pancreatic tissue sections were deparaffinized, rehydrated, and subjected to antigen retrieval. Endogenous peroxidase activity was blocked using hydrogen peroxide, followed by blocking with bovine serum albumin. The sections were incubated overnight at 4°C with a primary antibody against insulin (1:6400 dilution; 3014T, Cell Signaling Technology), followed by incubation with an appropriate HRP-conjugated secondary antibody. Immunoreactive signals were visualized using a DAB substrate, and the sections were counterstained with hematoxylin. All stained sections were observed using a Leica THUNDER microscope at the Biopolymer Research Center for Advanced Materials (BRCAM, Republic of Korea). Pancreatic insulin-positive areas were quantified from representative insulin-immunostained sections using ImageJ software under identical threshold settings and expressed relative to the NC group.

### Western Blotting Assay

Proteins were extracted from cultured cells or mouse skeletal muscle tissue using cell lysis buffer (Cell Signaling Technology), and protein concentrations were determined using the Pierce BCA Protein Assay Kit (Thermo Fisher Scientific). Equal amounts of protein were separated by 8% SDS-PAGE and transferred onto polyvinylidene fluoride membranes using a semi-dry transfer system (ATTO, Japan). Membranes were blocked with bovine serum albumin (BSA) for 1 h and incubated overnight at 4°C with primary antibodies against target proteins, followed by incubation with HRP-conjugated secondary antibodies for 40 min. Protein bands were visualized using a chemiluminescence detection system (LuminoGraph 3 Lite, ATTO, Japan). Band intensities were quantified using ImageJ software. Phosphorylated AKT and AMPK were expressed as ratios relative to their corresponding total protein levels, whereas GLUT4 and ADIPOR1 were normalized to vinculin. Densitometric quantification was performed using skeletal muscle samples from n = 3 biological replicates per group.

### Statistical Analysis

All statistical analyses were performed using GraphPad Prism software (version 10.5, GraphPad Software, USA). Data are presented as mean ± SD. Differences among groups were analyzed using one-way or two-way analysis of variance (ANOVA), depending on the experimental design, followed by Dunnett’s or Tukey’s multiple comparisons test. Repeated measures two-way ANOVA were applied for time-course analyses where appropriate. A *p*-value of less than 0.05 was considered statistically significant.

## Results

### *In Vitro* Anti-Diabetic Activity And Metabolic Characterization of *B. breve* B-3

**Fig. 1:**
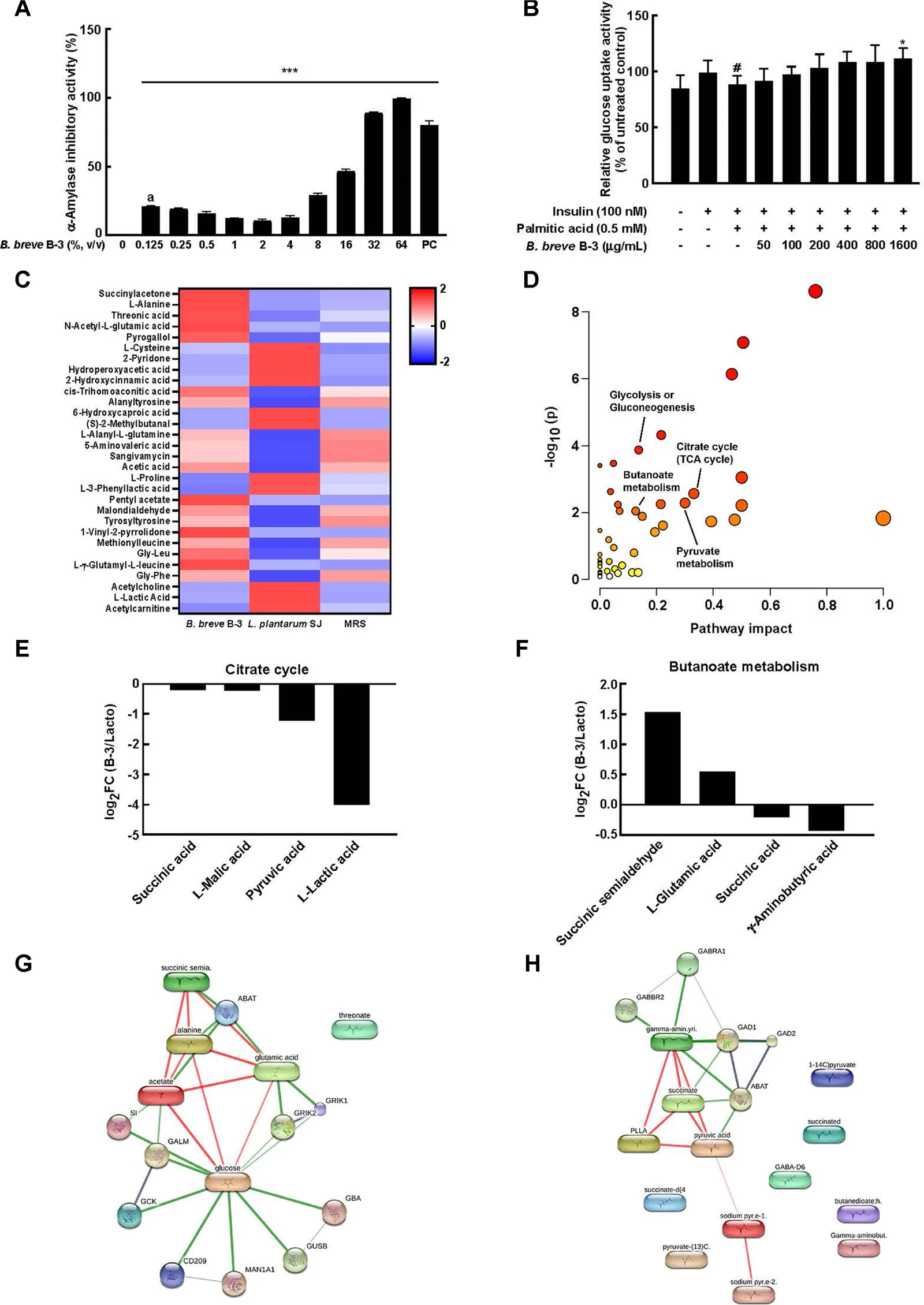
In vitro anti-diabetic activity and strain-associated metabolomic profiling of *Bifidobacterium breve* B-3. (**A**) α-Amylase inhibitory activity of *B. breve* B-3 cell-free culture supernatant (**CFS**) at the indicated CFS levels. PC indicates acarbose as a positive control. (**B**) Effects of *B. breve* B-3 on insulin-stimulated glucose uptake in palmitic acid-induced insulin-resistant C2C12 myotubes. Glucose uptake was assessed using 2-NBDG and expressed as a percentage of the untreated control. (**C**) Heatmap analysis of differentially abundant metabolites in culture supernatants of *B. breve* B-3, *L. plantarum* SJ, and MRS medium. (**D**) Pathway enrichment analysis of differentially abundant metabolites. (**E, F**) Log_2_ fold change values of representative metabolites involved in citrate cycle and butanoate metabolism pathways (B-3 vs. Lacto). (**G, H**) STITCH-based predicted metabolite interaction networks derived from *B. breve* B-3 and *L. plantarum* SJ enriched metabolites. Data are presented as mean ± SD. Statistical significance was determined by one-way ANOVA followed by Dunnett’s multiple comparisons test. For panel A, ***p < 0.001 compared with the enzyme control group. For panel B, ^#^*p* < 0.05, compared with the insulin-treated control group; *p < 0.05, compared with insulin and palmitic acid-treated group.

To evaluate the potential anti-diabetic activity of *B. breve* B-3, α-amylase inhibitory activity was first assessed. *B. breve* B-3 CFS increased α-amylase inhibitory activity in a CFS level-dependent manner, and higher CFS levels showed inhibitory activity comparable to that of the positive control (acarbose) (Fig. 1A). Next, the effect of lyophilized *B. breve* B-3 on cellular glucose uptake was examined in palmitic acid-induced insulin-resistant C2C12 myotubes. Palmitic acid treatment significantly reduced insulin-stimulated glucose uptake compared with insulin-treated control cells. However, treatment with *B. breve* B-3 increased glucose uptake, with the highest concentration showing a significant improvement compared with the palmitic acid-treated group (Fig. 1B). To further characterize metabolic differences between probiotic strains, LC–MS/MS-based metabolomic profiling was performed. Heatmap analysis revealed distinct metabolite abundance patterns among *B. breve* B-3, *L. plantarum* SJ, and MRS medium (Fig. 1C). Pathway enrichment analysis of differentially abundant metabolites identified several pathways related to energy metabolism, including glycolysis/gluconeogenesis, the citrate cycle (TCA cycle), pyruvate metabolism, and butanoate metabolism (Fig. 1D). Within the citrate cycle pathway, pyruvic acid and L-lactic acid showed negative log2 fold change values in *B. breve* B-3 compared with *L. plantarum* SJ, whereas L-malic acid and succinic acid exhibited relatively smaller differences between the two strains (Fig. 1E). In the butanoate metabolism pathway, succinic semialdehyde and L-glutamic acid displayed positive log2 fold change values in *B. breve* B-3 relative to *L. plantarum* SJ, whereas γ-aminobutyric acid and succinic acid were reduced (Fig. 1F). To further explore functional differences between probiotic strains, differentially abundant metabolites were subjected to STITCH-based interaction network analysis. The predicted metabolite–protein interaction networks derived from *B. breve* B-3-enriched metabolites differed from those derived from *L. plantarum* SJ (Fig. 1G and H), suggesting distinct metabolite-associated interaction patterns between the two probiotic strains. Collectively, these findings demonstrate that *B. breve* B-3 exhibits *in vitro* anti-diabetic activity and possesses a metabolic profile distinct from that of the comparative probiotic strain.

### Effects of *Bifidobacterium breve* B-3 on Body Weight and Glycemic Parameters

**Fig. 2:**
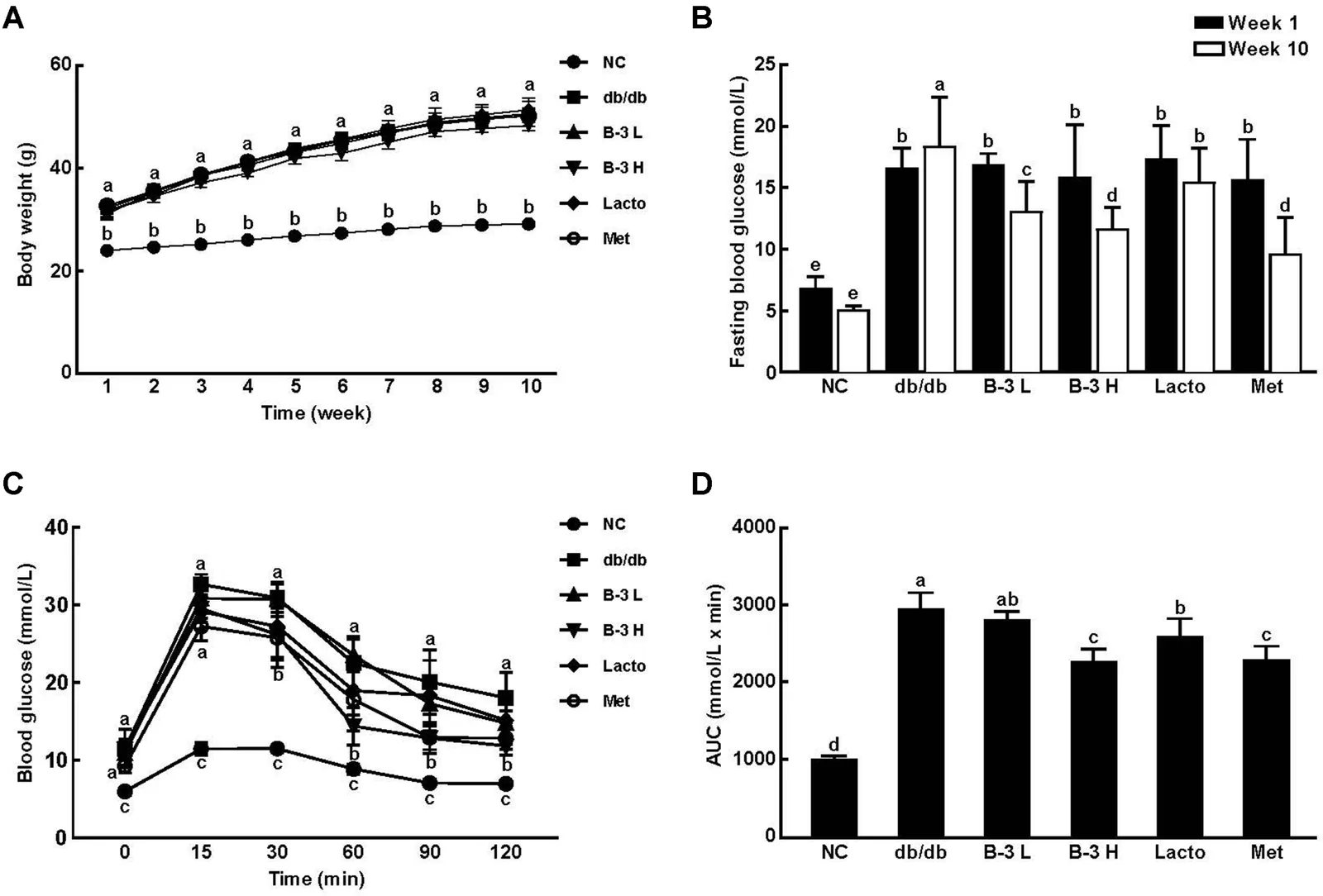
Effects of *Bifidobacterium breve* B-3 on body weight and glycemic parameters in db/db mice. (**A**) Body weight changes during the experimental period. (**B**) Fasting blood glucose levels at week 1 and week 10. (**C**) Oral glucose tolerance test (**OGTT**) curves showing blood glucose levels at indicated time points (0, 15, 30, 60, 90, and 120 min) following oral glucose administration. (**D**) Area under the curve of the OGTT. Data are presented as mean ± SD. Statistical significance was determined by two-way ANOVA followed by Tukey’s multiple comparisons test. Different letters indicate statistically significant differences among groups (*p* < 0.05).

We first investigated body weight and glycemic parameters in db/db mice following oral administration of *B. breve* B-3. Body weight was significantly higher in the db/db group than in the NC group throughout the experimental period. However, no significant differences in body weight were observed between the db/db group and the treatment groups (Fig. 2A). Weekly food intake, water intake, and feed efficiency ratio (FER) were monitored throughout the intervention period (Fig. S2). Although the db/db group exhibited higher food and water intake than the NC group, no significant differences were observed between the db/db and *B. breve* B-3-treated groups. Similarly, FER was elevated in the db/db group compared with the NC group, whereas *B. breve* B-3 treatment did not significantly alter FER. These findings indicate that the glycemic effects of *B. breve* B-3 were not attributable to changes in food intake, water intake, or feed efficiency. After 10 weeks of intervention, fasting blood glucose levels significantly increased in the db/db group relative to baseline, whereas glucose levels in the B-3-treated groups remained relatively stable during the intervention period (Fig. 2B). To further evaluate glucose tolerance, an OGTT was performed. Both the B-3 H and Met groups showed significantly improved glucose tolerance starting at 30 min after glucose loading compared with the db/db group (Fig. 2C). Consistently, AUC analysis of the OGTT further supported this improvement (Fig. 2D). In contrast, the *L. plantarum* SJ group showed no significant changes in glucose-related parameters compared with the db/db group. Collectively, these findings indicate that *B. breve* B-3 improved glucose tolerance in the db/db group without significantly affecting body weight, food intake, or feed efficiency.

### *Bifidobacterium breve* B-3 Regulates Incretin Hormones and Insulin-Related Parameters

**Fig. 3:**
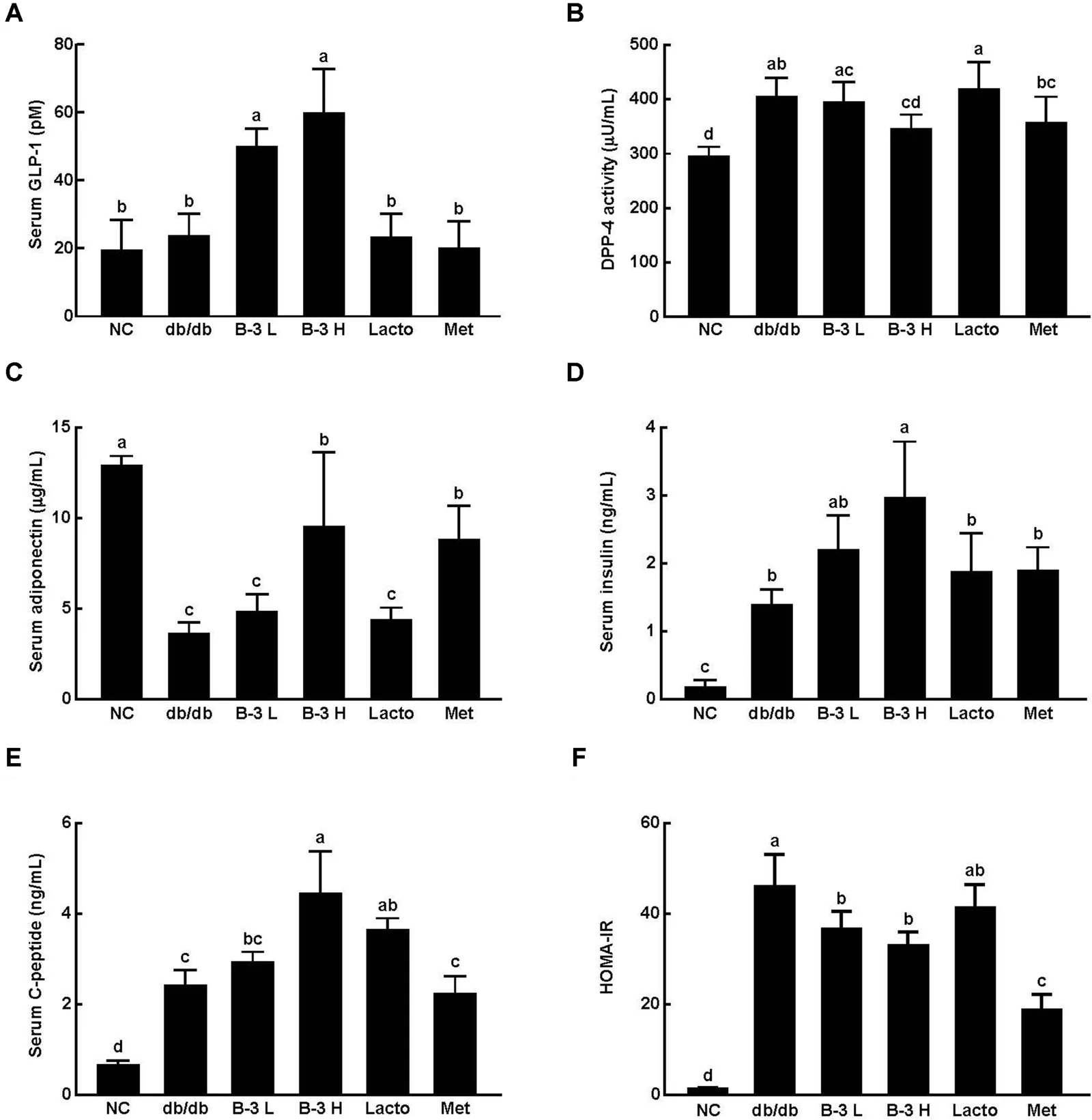
Effects of *Bifidobacterium breve* B-3 on incretin hormones and insulin-related parameters. (**A**) Serum GLP-1 levels. (**B**) Serum DPP-4 activity. (**C**) Serum adiponectin levels. (**D**) Serum insulin levels. (**E**) Serum C-peptide levels. (**F**) HOMA-IR. Data are presented as mean ± SD. Statistical significance was determined by one-way ANOVA followed by Tukey’s multiple comparisons test. Different letters indicate significant differences among groups (*p* < 0.05).

Given that *B. breve* B-3 improved glycemic parameters, we next investigated whether these effects were associated with alterations in incretin hormones and insulin-related parameters. Serum GLP-1 levels did not differ significantly between the NC and db/db groups. However, *B. breve* B-3 administration significantly increased serum GLP-1 levels compared with the db/db group (Fig. 3A). Because GLP-1 is rapidly degraded by DPP-4 [34], DPP-4 activity was additionally evaluated. DPP-4 activity was significantly elevated in the db/db group, whereas B-3 H significantly attenuated DPP-4 activity (Fig. 3B). Furthermore, serum adiponectin levels were significantly increased in the B-3 H group (Fig. 3C). Along with these changes, serum insulin levels were significantly increased in the *B. breve* B-3 treated groups compared with the db/db group (Fig. 3D). Serum C-peptide levels were also increased following *B. breve* B-3 administration, with a more pronounced increase compared with the *L. plantarum* SJ group (Fig. 3E). To further assess insulin resistance, HOMA-IR was calculated. HOMA-IR values decreased in the *B. breve* B-3 treated groups compared with the db/db group (Fig. 3F). In contrast, under the present experimental conditions, the *L. plantarum* SJ group showed limited changes in most endocrine and glucose-related parameters compared with the *B. breve* B-3–treated groups. Collectively, these results suggest that *B. breve* B-3 modulates incretin-related endocrine parameters and supports insulin-secretory responses in db/db mice.

### *Bifidobacterium breve* B-3 Modulates Intestinal Glucose Transporters, SCFA Receptors, and Intestinal Morphology

**Fig. 4:**
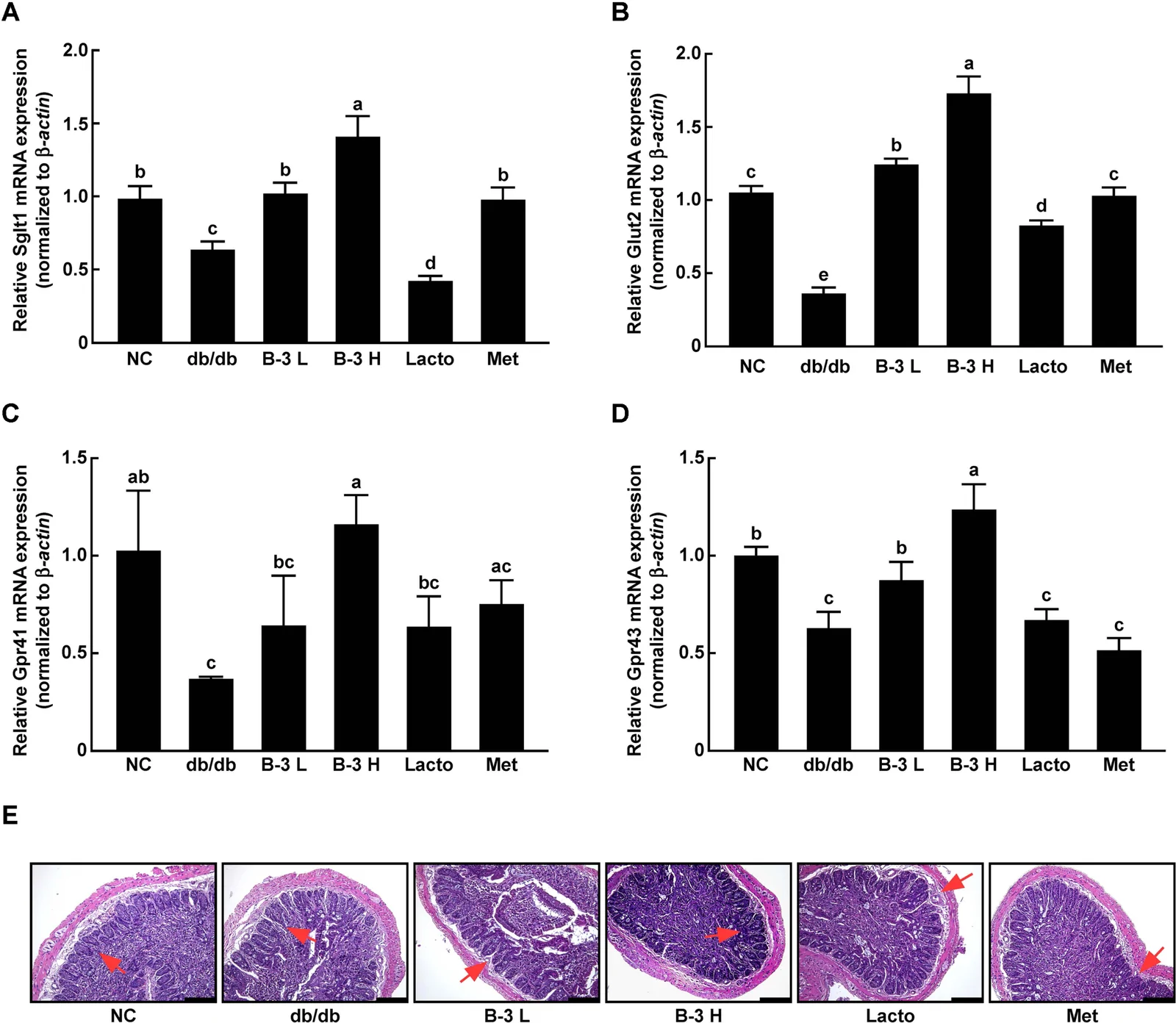
Effects of *Bifidobacterium breve* B-3 on intestinal glucose transporters, SCFA receptors, and intestinal morphology. (**A, B**) Relative mRNA expression levels of intestinal glucose transporters *Sglt1* and *Glut2*. (**C, D**) Relative mRNA expression levels of SCFA receptors *Gpr41* and *Gpr43*. (**E**) Representative images of small intestinal tissue stained with H&E. Red arrows indicate representative areas of intestinal mucosal morphology. Scale bar = 100 μm. Data are presented as mean ± SD. Statistical significance was determined by one-way ANOVA followed by Tukey’s multiple comparisons test. Different letters indicate significant differences among groups (*p* < 0.05).

To examine intestinal factors associated with the metabolic effects of *B. breve* B-3, we investigated alterations in intestinal glucose transporters, SCFA receptors, and intestinal morphology. The mRNA expression levels of *Sglt1* and *Glut2* in the small intestine were significantly reduced in the db/db group compared with the NC group. However, *B. breve* B-3 administration significantly increased the expression levels of *Sglt1* and *Glut2* compared with the db/db group (Fig. 4A and B). In addition, the mRNA expression levels of SCFA receptors, *Gpr41* and *Gpr43*, were significantly decreased in the db/db group, whereas *B. breve* B-3 treatment significantly increased the expression of both receptors (Fig. 4C and D). Histological analysis of the small intestine revealed modest alterations in villus morphology in the db/db group compared with the NC group. In contrast, *B. breve* B-3–treated groups showed relatively preserved villus morphology, as observed by H&E staining (Fig. 4E). By comparison, the *L. plantarum* SJ group showed limited changes in the expression of intestinal glucose transporters and SCFA receptors and did not exhibit comparable histological features. Collectively, these findings indicate that *B. breve* B-3 administration was associated with altered intestinal glucose transporters and SCFA receptor expression, along with changes in villus morphology in db/db mice.

### *Bifidobacterium breve* B-3 Modulates Insulin Signaling–Related Proteins in Skeletal Muscle

**Fig. 5:**
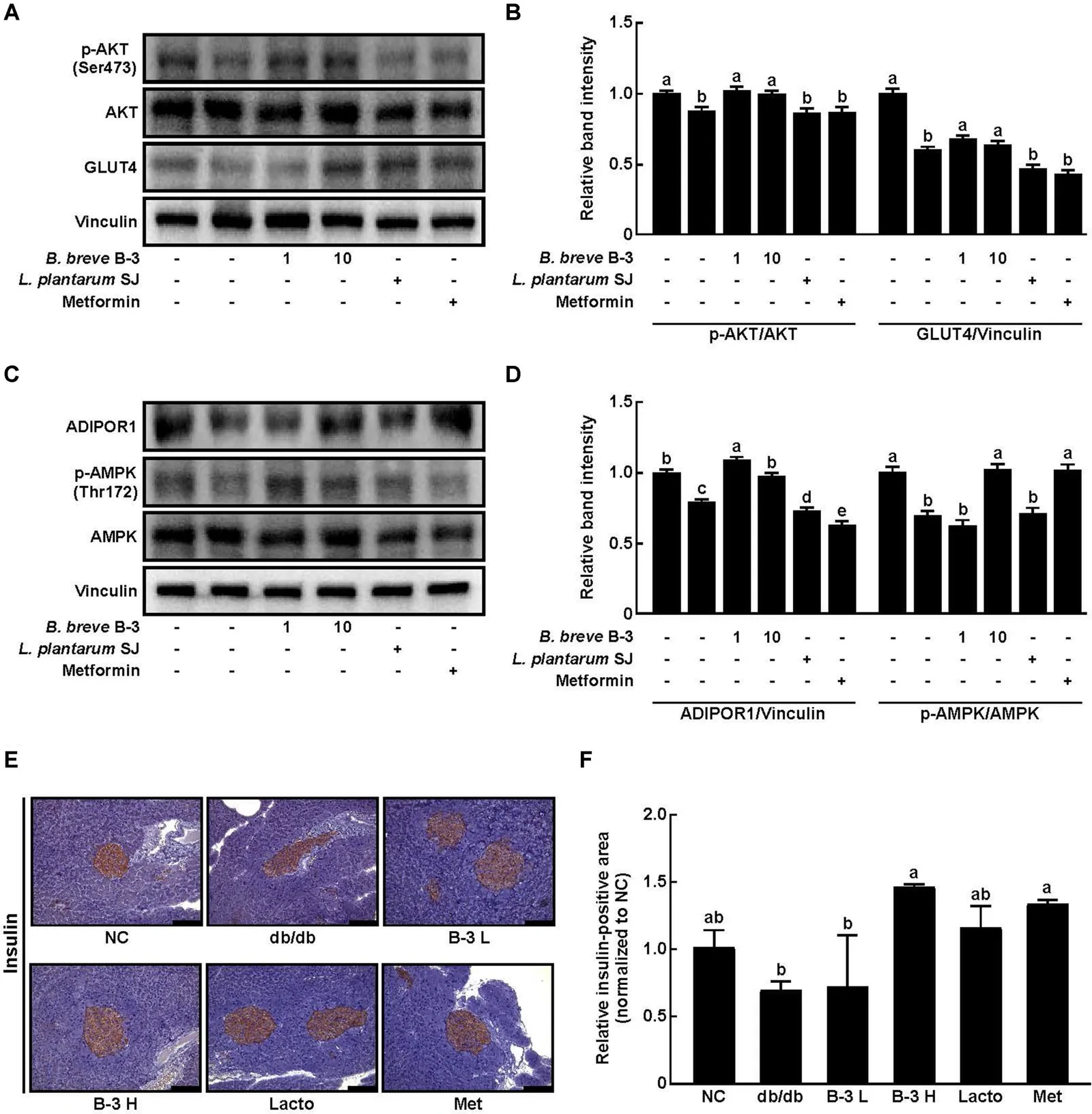
Effects of *Bifidobacterium breve* B-3 on insulin signaling-related proteins in skeletal muscle and pancreatic insulin immunoreactivity. (**A**) Representative immunoblot images of p-AKT, AKT, GLUT4, and vinculin. (**B**) Quantification of p-AKT/AKT and GLUT4/vinculin. (**C**) Representative immunoblot images of ADIPOR1, p-AMPK, AMPK, and vinculin. (**D**) Quantification of ADIPOR1/vinculin and p-AMPK/AMPK. (**E**) Representative pancreatic sections immunostained for insulin. (**F**) Quantification of insulin-positive area. Band intensities were quantified from n = 3 biological replicates per group. Data are presented as mean ± SD. Statistical significance was determined by one-way ANOVA followed by Tukey’s multiple comparisons test. Different letters indicate significant differences among groups (*p* < 0.05).

To investigate whether *B. breve* B-3 affected peripheral insulin signaling, AKT/GLUT4 and ADIPOR1/AMPK related proteins were analyzed in skeletal muscle. Representative immunoblot images showed changes in AKT/GLUT4 related proteins following *B. breve* B-3 administration, and densitometric quantification showed increased p-AKT/AKT ratio and GLUT4 expression compared with the db/db group (Fig. 5A and 5B). Similarly, *B. breve* B-3 administration was associated with increased ADIPOR1 expression and p-AMPK/AMPK ratio, as shown by representative immunoblotting and densitometric quantification (Fig. 5C and 5D). Pancreatic insulin immunoreactivity was also evaluated by immunohistochemistry, and quantitative analysis showed that the insulin-positive area was reduced in the db/db group and partially increased in the *B. breve* B-3 high-dose group (Fig. 5E and 5F).

## Discussion

T2DM is characterized by chronic hyperglycemia driven by impaired insulin secretion and peripheral insulin resistance, with emerging evidence highlighting the role of gut-derived metabolic signals in systemic glucose regulation [10, 12]. In the present study, we investigated whether *B. breve* B-3 possesses distinct metabolic characteristics relevant to glucose homeostasis and whether these properties translate into functional benefits in a diabetic model.

Initial *in vitro* analyses demonstrated that *B. breve* B-3 exerted concentration-dependent α-amylase inhibitory activity and enhanced insulin-stimulated glucose uptake in palmitic acid-induced insulin-resistant C2C12 myotubes (Fig. 1A and 1B). In addition, *B. breve* B-3 exhibited a metabolic profile distinct from that of *Lactobacillus plantarum* SJ (Fig. 1C). LC–MS/MS analysis revealed differential enrichment of glycolysis/gluconeogenesis, the citrate cycle, pyruvate metabolism, and butanoate metabolism pathways (Fig. 1D–F). Given that *Bifidobacterium* species utilize the fructose-6-phosphate phosphoketolase pathway [35], these findings suggest differences in carbon metabolism compared with the classical lactic acid fermentation pathways of *Lactobacillus* species [36]. Furthermore, STITCH-based network analysis revealed divergent metabolite–protein connectivity patterns between the two strains (Fig. 1G and H). While *L. plantarum* SJ-enriched metabolites were primarily linked to GABA-related pathways, *B. breve* B-3-associated metabolites showed greater connectivity with nodes related to central carbon metabolism and the tricarboxylic acid (TCA) cycle. Although predictive, these observations suggest that *B. breve* B-3 may generate metabolite environments distinct from those of the comparative probiotic strain and potentially relevant to host metabolic regulation.

Consistent with these observations, *B. breve* B-3 administration improved fasting blood glucose levels and glucose tolerance in db/db mice without affecting body weight or energy intake (Fig. 2). These findings suggest that the glucose-lowering effects of *B. breve* B-3 were not secondary to reduced caloric intake or weight loss. In addition, serum ALT, AST, and ALP levels were not increased following *B. breve* B-3 administration compared with the db/db group, suggesting no overt elevation of hepatic enzyme markers under the present experimental conditions (Fig. S1). *B. breve* B-3 was also associated with modulation of incretin-related endocrine parameters, as evidenced by increased circulating GLP-1 levels and reduced DPP-4 activity [34, 37]. In parallel, serum insulin and C-peptide levels were increased following *B. breve* B-3 administration. Because db/db mice exhibit compensatory hyperinsulinemia during diabetes progression, these increases should be interpreted cautiously and do not by themselves establish improved insulin sensitivity [38]. The observed changes may reflect maintained or supported insulin-secretory capacity under diabetic stress, but they do not by themselves establish improved insulin sensitivity. Together with increased adiponectin levels and reduced HOMA-IR values, these findings indicate changes in fasting glucose–insulin homeostasis (Fig. 3). However, HOMA-IR is an indirect index, and its interpretation in leptin receptor-deficient db/db mice may be limited.

Although short-chain fatty acids were not directly quantified in the present study, *B. breve* B-3 treatment was associated with changes in the intestinal expression of the SCFA receptors *Gpr41* and *Gpr43*, together with changes in the glucose transporters *Sglt1* and *Glut2* (Fig. 4A–D). Acetate, a major metabolite produced by *Bifidobacterium* species, has been reported to activate GPR43 and stimulate GLP-1 secretion [17, 39]. SGLT1 and GLUT2 are key intestinal glucose transporters involved in glucose absorption, and SGLT1 has also been implicated in glucose-dependent incretin secretion [13]. Therefore, these changes should be interpreted as alterations in intestinal signaling and glucose-handling markers rather than direct evidence of altered glucose absorptive flux. Direct in vivo metabolite quantification, gut microbiota analysis, and functional glucose absorption studies will be required to validate this proposed route. Histological examination of the small intestine revealed modest alterations in villus morphology in db/db mice, whereas *B. breve* B-3-treated groups showed relatively preserved villus morphology compared with the db/db group (Fig. 4E). Together, these findings suggest that *B. breve* B-3 may influence intestinal glucose-related signaling and intestinal morphology under diabetic conditions.

Peripheral insulin signaling-related responses were also altered following *B. breve* B-3 administration. In skeletal muscle, *B. breve* B-3 increased insulin-stimulated AKT phosphorylation and GLUT4 expression compared with the db/db group (Fig. 5A and 5B). Because AKT signaling is closely associated with GLUT4-mediated glucose transport in skeletal muscle [40, 41], these findings suggest altered insulin signaling-related responses in peripheral tissues. In addition, *B. breve* B-3 increased ADIPOR1 expression and AMPK phosphorylation (Fig. 5C and 5D), consistent with modulation of adiponectin-related metabolic signaling [42, 43]. Pancreatic insulin immunohistochemical analysis showed reduced insulin-positive area in the db/db group, whereas *B. breve* B-3 high-dose treatment group significantly increased insulin immunoreactivity compared with the db/db group (Fig. 5E and 5F). Given that pancreatic islet disorganization is a recognized feature of T2DM progression [44, 45], the partial preservation of pancreatic insulin immunoreactivity may reflect attenuation of diabetes-associated pancreatic dysfunction. Collectively, these findings suggest that *B. breve* B-3 improved glucose homeostasis in db/db mice in association with changes in incretin-related endocrine parameters, intestinal glucose related markers, and peripheral insulin signaling associated responses. Further studies incorporating direct short-chain fatty acid quantification, gut microbiota analysis, and detailed metabolite validation will be necessary to clarify the relationship between microbial metabolites and host glucose regulation. *B. breve* B-3 improved glycemic control in db/db mice and was associated with incretin-related endocrine parameters, insulin-secretory responses, and glucose-related signaling responses. Compared with *L. plantarum* SJ under the present experimental conditions, *B. breve* B-3 showed greater effects on selected glucose-related parameters. However, because only one comparator strain was used, these results do not establish broad strain-specific superiority. The effects of B-3 were also endpoint-dependent, with some parameters showing clearer responses only at the high dose. Further studies using additional comparator strains are needed to validate the strain-associated characteristics of B-3.

## Supplemental Materials

Supplementary data for this paper are available on-line only at http://jmb.or.kr.



## Figures and Tables

**Table 1:** Primer sequences list for quantitative real-time PCR.

Primer name	Organism	Primer sequence (5'→3')	Accession ID
β-actin	Mus musculus	F: TCCATCATGAAGTGTGACGT R: TACTCCTGCTTGCTGATCCAC	NM_007393.5
Sglt1	Mus musculus	F: ACGGTATTATCCTGCTGGCG R: TCCCCTTGCCACTACAGAGT	NM_019810.4
Glut2	Mus musculus	F: ACCGGAATGTTCTTAGCCCC R: GCTGTACGCAAAACCCGAAG	NM_031197.2
Gpr41	Mus musculus	F: TGCTCATCTTCTTCGTCTGCTTCG R: GTCGGCTTGGAACTTGGAGGAG	NM_001033316.2
Gpr43	Mus musculus	F: CTGGCACAGTTCCTTGATCCTCAC R: GGCAGCAGCAGCAACAGGAG	NM_146187.4
