# Supplementary material for: Effects of Bifidobacterium breve B-3 on Glycemic Control and Glucose-Regulatory Responses in db/db Mice
Source: J Microbiol Biotechnol. 2026 Aug 18;36:e2605040. doi: 10.4014/jmb.2605.05040 (PMC13526894; doi:10.4014/jmb.2605.05040)

Supplementary Figure 1.

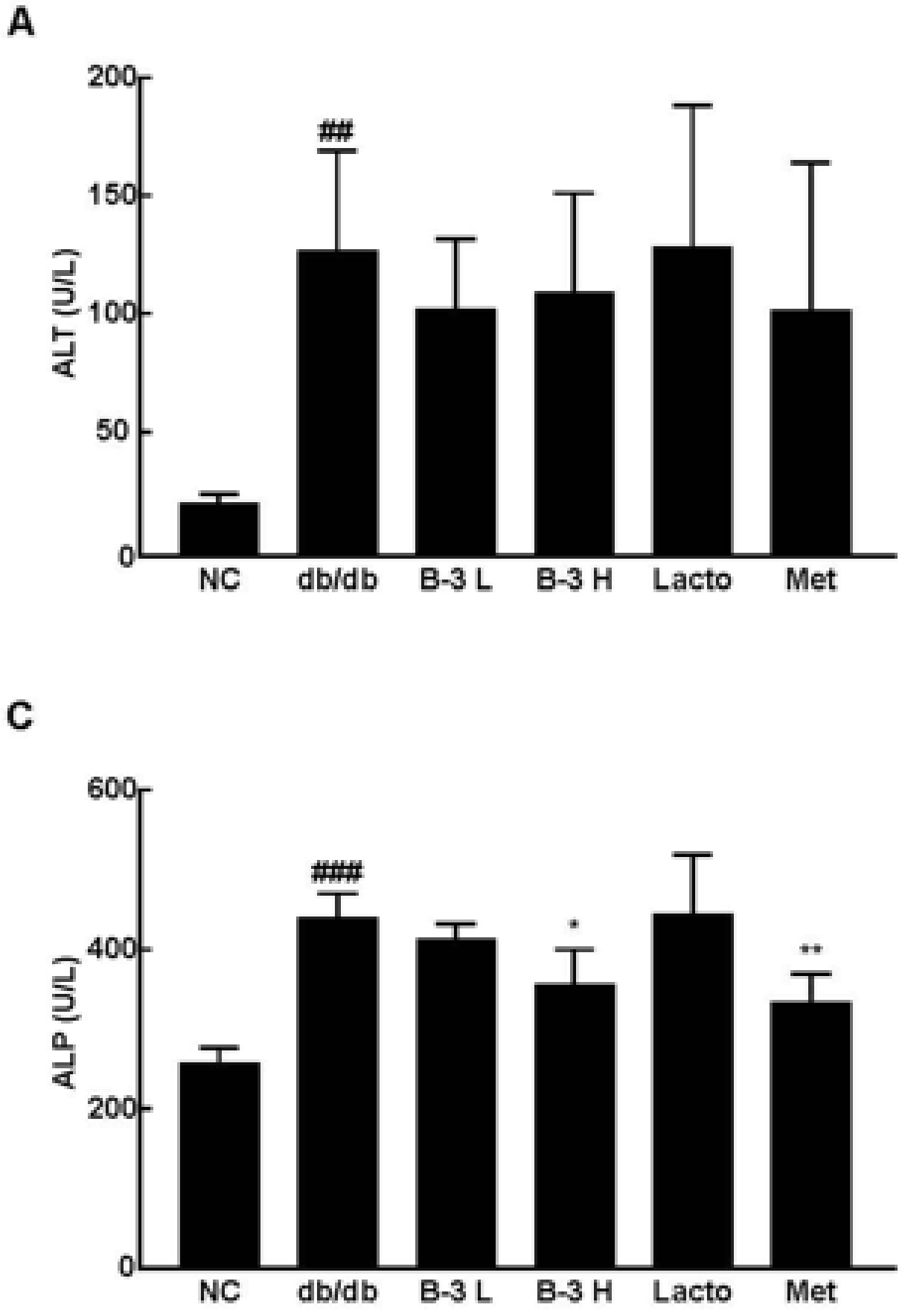

Supplementary Figure 2.

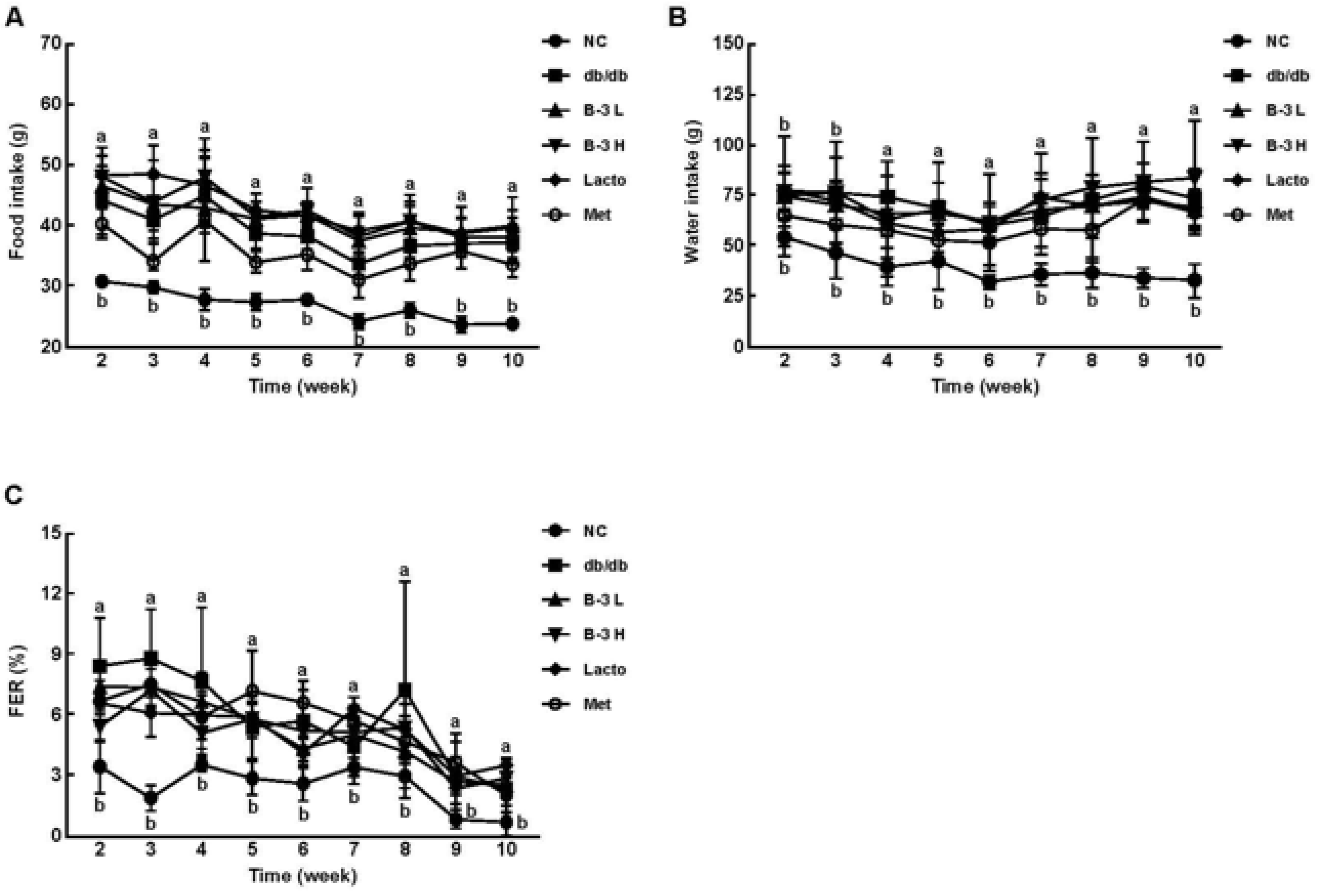

Supplement: Supplementary file 1 [file jmb-36-e2605040-supple.pdf]
